# The impact of morally injurious events in a refugee sample: A quantitative and qualitative study

**DOI:** 10.3389/fpsyt.2022.904808

**Published:** 2022-09-08

**Authors:** Nora Mooren, Paul A. Boelen, Simone M. de la Rie

**Affiliations:** ^1^ARQ National Psychotrauma Centre, Diemen, Netherlands; ^2^Department of Clinical Psychology, Faculty of Social Sciences, Utrecht University, Utrecht, Netherlands

**Keywords:** moral injury, moral stress, refugees, PTSD, guilt

## Abstract

**Background:**

Posttraumatic Stress Disorder (PTSD) is often reported by refugees that faced violence and persecution. Some stressful events may also entail moral conflicts or dilemmas, described as “potentially morally injurious events” (PMIE). Very few studies have yet investigated the nature of these PMIEs in traumatized refugees, using both quantitative and qualitative data.

**Method:**

For this retrospective study, secondary data analysis was used to examine the traumatic events of 183 patients. Based on established definitions of a PMIE, participants were allocated to a Moral Injury (MI) group if they reported lasting distress after exposure to an event of which they indicated that it transgressed their moral beliefs. The remaining participants were allocated to the No-MI group. The type of PMIEs was categorized using qualitative analysis. The groups were compared in terms of PTSD severity, feelings of guilt, and general mental health symptoms.

**Results:**

Of the total sample, 55 participants reported one or more acts of transgression (MI group) and 128 reported no acts of transgression (No-MI group). Analyses of PMIEs revealed six themes 1) failing to prevent harm to others, 2) not giving aid to people in need, 3) leaving family members behind that consequently lead to injury or death of others, 4) making indirect and direct moral decisions leading to injury or death of others, 5) betrayal, and 6) engaging in the harm of others. No differences were found between groups on the clinical outcomes, except for feelings of guilt.

**Conclusion:**

A considerable number of traumatized refugees reported confrontation with PMIEs. Experiencing PMIEs appeared unrelated to elevated posttraumatic mental health issues.

## Introduction

As a result of persecution, conflict, violence, and human rights violations, more than 89.3 million people worldwide were forcibly displaced of which 52.3 million internally displaced at the end of 2021 (1). The majority of the refugees has experienced multiple traumatic events such as sexual violence and imprisonment (2). Furthermore, many of them endured stressors during the migration process, such as separation from family, stays in refugee camps, and lengthy asylum procedures (3). Not surprisingly, the prevalence of mental health problems in refugees is high (4, 5). Mood and anxiety disorders are often reported, even years after resettlement (6, 7), indicating a high and persisting mental burden in refugees. The effects of violence and persecution go beyond fear-related reactions. Some traumatic experiences also entail moral conflicts or dilemmas and may be described as “potentially morally injurious events” (PMIEs). These events include “bearing witness to perceived immoral acts, failure to stop such actions, or perpetration of immoral acts that are inhumane, cruel, depraved, or violent, bringing about pain, suffering, or death of others” [(8), p. 9]. The term “moral injury” refers to “the lasting psychological, biological, spiritual, behavioral, and social impact of perpetrating, failing to prevent, or bearing witness to acts that transgress deeply held moral beliefs and expectations” [(9), p. 697]. Both definitions illustrate that moral injury and PMIEs can result from either active acts of commission (hereafter indicated as “commissions”) or a failure of acts that prevented harm (hereafter indicated as “omissions”) and can either be a result of appraisals of one's own moral transgressions (MI-Self) or appraisals of moral transgressions by others (MI-Other) (10, 11). There are indications that MI-self appraisals result in different outcomes than MI-other appraisals. For instance, research has shown that MI-Other appraisals were associated with more severe Posttraumatic Stress Disorder (PTSD) symptoms and MI-Self appraisals were associated with a lower level of intrusions (10, 11). In first-responder populations, it was found that actively perpetrating acts that transgressed moral values or beliefs (MI-self) was related to more self-blame, guilt and re-experiencing than exposure to life-threat situations without moral transgressions (12). Also, there was a reciprocal relationship between PMIEs with transgressions of oneself and PTSD symptoms 6 months later (13). Also, veterans who actively killed others had more suicidal ideations than individuals without these killing experiences (14).

Many studies investigating moral injury have focused on military populations (8, 15). Transgressions of moral beliefs included events such as killing, betrayal, and failing to prevent harm to civilians (16). Studies in a refugee population sample demonstrated that the majority of the refugees reported MI-other appraisals or a combination of MI-other and MI-self appraisals. Also, being troubled by acts of moral transgression was related to mental health problems such as PTSD, depression, and anger (10, 11, 17). Moral transgressions also predicted externalizing symptoms but not internalizing symptoms in refugee adolescents (18). Results provided evidence that the majority in a help-seeking refugee sample (68) reported embitterment and moral injury appraisals were positively associated with levels of embitterment, revealing the importance of perceived injustice in mental health problems after trauma exposure (19). Still there is very limited knowledge on the prevalence of moral injury in refugees as well as the nature of the PMIEs that refugees endure. Refugees are often exposed to a cumulation of traumatic events, that may meet the definition of a PMIE (8), but the nature and scope of PMIEs in refugees is yet to be examined. Also, little is known about the relationship between moral transgressions (either by oneself or others) and feelings of guilt, PTSD symptoms, and other mental health symptoms in refugees (12–14).

As far as we are concerned, there are no studies that examined PMIEs in a refugee sample by describing the nature of the PMIEs that refugees report. Therefore, the first aim of this study was to examine the nature of PMIEs among refugees, using a qualitative approach. Whereas we expected to find similar PMIEs as found in military contexts, we assumed that a number of PMIEs may reflect moral transgressions that are typical for a refugee population. For instance, leaving family members behind or making decisions about who receives (medical) aid first. Next to the PMIEs, we also aimed to provide descriptions of the traumatic events that patients were exposed to, differentiating between MI-self and MI-other and report the number of events in the total group of patients. Our second aim was to compare refugees with and without PMIEs in terms of PTSD severity, feelings of guilt, and general mental health complaints. We expected participants in the MI group to have more severe psychological complaints, manifesting in higher levels of PTSD, feelings of guilt, and other mental health complaints. This prediction was based on earlier studies (4, 10, 20, 21). In specific, we expected that the MI group would report more cluster D symptoms and feelings of guilt than the No-MI group since moral transgressions are associated with more feelings of guilt and wrongdoing (22). Guilt is often seen as an important emotion in moral injury (23) and can be viewed as a central component of PMIEs. Guilt is associated with having committed a moral transgression (MI-Self), whereas MI-Other events have been associated with anger in refugee populations (10). In a recent study among refugees, it was found that both preexisting general moral beliefs and situation specific blame appraisals were important for emotional outcomes such as guilt and anger (24).

## Method

### Participants

This retrospective study was conducted at a Dutch center for specialist diagnostics and treatment of people with complex psychotrauma complaints (i.e., ARQ National Psychotrauma Centre/Centrum'45). The majority of patients referred to this centre are severely traumatized individuals who received one or multiple treatments at other institutions, with limited success. The sample in the current study consisted of refugees (all above 18 years old) referred for diagnostics and treatment between 2014 and 2018.

### Procedure

Data for this retrospective study were primarily collected for clinical purposes as part of the routine screening and assessment procedure prior to the start of treatment at ARQ Centrum'45. Data that were not stored automatically were entered into the system by authorized members of the clinical staff. Subsequently, data were archived anonymously for scientific research purposes by our data management department. After this procedure, anonymized data were made available to the researchers conducting this retrospective study. Patients were informed about the storage of anonymized assessment data and given the opportunity to have their data removed from the database, a procedure that is coordinated by our data management department. At intake patients were interviewed about their psychological complaints and the traumatic events they encountered. They also filled out several questionnaires as part of the Routine Outcome Monitoring, including the Brief Symptom Inventory (BSI), and the Life Events Checklist for DSM-5 (LEC-5). We used officially translated questionnaires in several languages (e.g., Dutch, English, French, Farsi, Bosnian Serbian, and Arabic) and if a specific language was not available, an official interpreter assisted. Furthermore the Clinician-Administered PTSD Scale for DSM-5 (CAPS-5) was administered in English, Dutch or with assistance of an interpreter. Patients were asked to offer written informed consent that the data from the assessment procedure as well as their electronical patient file could anonymously be archived for scientific research purposes; 379 patients did so.

For secondary analysis, participants were allocated into two groups based on information of the intake procedure: the MI group (one or more PMIEs) or no-MI group (no PMIEs). The traumatic events reported at intake were examined in order to assign the group categorization. First, one clinician made a broad preselection of the intake reports in order to categorize the events that were mentioned in the reports as “potential MIE.” Traumatic events were indicated as PMIE when the description of the event and its consequences included information on 1) moral transgressions of the person himself or others (e.g., “watching how a friend was physical attacked”), or 2) the event was accompanied by feelings of guilt, shame, regret, remorse (e.g., “felt guilty because I didn't react to it”), or 3) the event included a perceived moral decision or a moral conflict directly related to the event (e.g., “I made the choice to flee but wasn't sure about it”). The potential PMIEs were listed separately in an anonymized file. In case more than one PMIE was reported in the intake report, all PMIEs were selected. Then, two other clinicians categorized the PMIEs following the definition of PMIEs by Drescher et al. (8) and the definition of moral injury by Litz et al. (9): An event was categorized as PMIE when the description of the event included 1) either a moral decision or a moral transgression by the person himself or others (either commissions or omissions) and 2) negative (emotional) consequences for the person himself or important others, either in a psychological, biological, spiritual, behavioral, or social manner. All participants with a designated PMIE were allocated to the MI group. When no information on the traumatic events or moral transgressions could be found, the information was ambiguous, or there was no information on the consequences of the event, patients were excluded from the current study, resulting in the reduction of the total sample of 379 participants to a total number of 183 participants. Of this sample 55 participants were assigned to the MI group and 128 participants to the No-MI group.

### Measures

#### Demographics

As part of the assessment procedure (described above) the following demographical variables were documented: age, gender, and country of origin.

#### Traumatic events

The Life Events Checklist for DSM-5 (LEC-5) is a 17-item self-report measure used to screen for exposure to potentially traumatic events, as defined with the A-criterion of PTSD according to the DSM-5 (25). It assesses exposure to 16 events known to lead to PTSD or distress and one appended item assessing any additional stressful event. Answers are rated on 6-point scales with anchors: 1 = “happened to me”; 2 = “witnessed it”; 3 = “learned about it”; 4 = “part of my job,” 5 = “not sure”; 6 = “does not apply.” Findings show the LEC is a psychometrically sound instrument (26).

#### PTSD severity

The Clinician-Administered PTSD scale for DSM-5 (CAPS-5) is a 30-item structured diagnostic interview that measures the number of PTSD symptoms (25) as well as PTSD severity and delayed expression. The CAPS-5 is a psychometrically sound measure, with strong reliability and validity (27, 28). The total severity score demonstrated high internal consistency (α = 0.88). The subscale in this study that measured criteria D symptoms of PTSD also showed good internal consistency (α = 0.76).

#### Mental health symptoms and feelings of guilt

The Brief Symptom Inventory (BSI) is a 53-item self-report questionnaire (29) that measures symptoms of psychological stress on nine subscales: depressive mood, interpersonal sensitivity, hostility, somatization, psychoticism, suspicion, phobic fear, cognitive problems and anxiety. One item of the BSI (“feelings of guilt”) was individually analyzed to assess guilt. Answers are scored on a 5-point Likert scale (0 = “totally disagree” to 4 “totally agree”). Researchers have found good psychometric properties of the instrument in the general (30) and refugee population (31).

### Statistical analysis

For this retrospective study, we used secondary data analysis. IBM SPSS Statistics 27.0 was used to conduct the statistical analyses, performed with a significance level of *p* <0.05 (two-tailed). The data were screened for multivariate and univariate outliers across and within conditions according to the procedure by Tabachnick and Fidell (32). There were no multivariate outliers detected with Mahalanobis distances. However, there were multiple univariate outliers (more than three standard deviations) on the variables trauma load (LEC-5 total score), PTSD severity (CAPS-5 total score), and criterion D symptoms (number of symptoms and severity). The outlier cases of these variables were replaced with the highest non-outlier case (32). Missing data were detected for the variable trauma load (*n* = 12) (measured with the LEC), for the PTSD severity variable (*n* = 1), and for the BSI total score (*n* = 1).

The assumptions of independence of observations and normality were met. However homogeneity of variances were not met for all variables. For the variables PTSD severity and criterion D symptoms (severity) the variance was significantly different in the two groups, *F*_(1, 180)_ = 11.36, *p* <0.001 and *F*_(1, 180)_ = 5.75, *p* <0.05, respectively. For these variables, the Welch *t*-test was used in the analyses. Due to unequal group sizes, Pillai's trace was used in the interpretation of the results as it is more robust than other statistics to violations of model assumptions (33).

For our fist aim, qualitative analysis was carried out. The PMIEs of the participants were categorized in themes based on coding of the events following an inductive approach. The two clinicians that made the final categorization of PMIEs also categorized all PMIEs into either; commissions or omissions. Also, they made a distinction between MI-self and MI-other. The distinction between self and other was based on the Moral Injury Events Scale (MIES) (16, 20) and the Moral Injury Appraisal Scale (MIAS) (11) where events were categorized as MI-self when the individual was the one who committed an act that was morally wrong or failed to prevent acts that were morally wrong (e.g., “I am troubled by morally wrong things I have done” and “I went against my own morals by failing to do something I should have done”) (MIAS). An event was categorized as MI-other when individuals were troubled because others acted morally wrong (e.g., “I am troubled because I saw other people do things that were morally wrong”) (MIAS). Lastly, the clinicians closely examined the summaries of the PMIEs in order to identify common themes following an inductive approach. This was done by highlighting the most important words or sentences that described the content and subsequently identify common themes. For instance, sentences such as “tried to give medical aid” and “was not able to help” were put together and labeled as the category “not giving aid to people in need.”

For our second aim, independent samples *t*-tests and a chi-square test were run to explore the differences between MI groups on the demographical variables. The differences between the MI groups (independent variable) on the dependent variables mental health symptoms (total score BSI), and feelings of guilt (sub-item of the BSI) were assessed with a multivariate analysis of variance (MANOVA). The differences between MI groups (independent variable) on PTSD severity and cluster D symptoms of PTSD were assessed with two Welch *t*-tests. Fisher's exact test (crosstabs) was used to test the differences between groups with respect to item 16 of the LEC-5 (“serious injury, harm, or death you caused to someone else”).

## Results

### Demographics

Table 1 shows the descriptive statistics of the demographic variables. The MI group and the No-MI group differed significantly with respect to gender, $\chi_{\left(1,\;183\right)}^{2}$ = 9.07, *p* = 0.003, but not with respect to age *F*_(1, 182)_ = 2.83, *p* = 0.094, η^2^ = 0.015. In the total sample, there were more male than female patients and there were only nine women in the MI-group in comparison to 50 women in the No-MI group. The age of participants ranged from 18 to 74 years. Participants were included from more than 47 different countries of origin. Most participants were from (fomer) Yugoslavia (13.1%), Afghanistan (10.9%), Iran (9.8%), Iraq (9.3%), Syria (7.1%), and Nigeria (6.6%). There were six participants of which the country of origin was not documented.

**Table 1:** Descriptive statistics of demographic variables for each group.

**Measure**		**Moral injury group**		**No-moral injury group**		**Total**	
		***N* (%)**	**M (SD)**	***N* (%)**	**M (SD)**	***N* (%)**	**M (SD)**
Total sample		55 (30)		128 (70)		183 (100)	
Age			39.78 (10.56)		42.78 (12.18)		40.68 (11.12)
Gender	Female	9 (15.3)		50 (84.7)		59 (32.2)	
	Male	46 (37.1)		78 (62.9)		124 (67.8)	
Country of origin	Afghanistan	7 (12.7)		7 (12.7)		20 (10.9)	
	Iran	5 (9.1)		13 (10.2)		18 (9.8)	
	Iraq	5 (9.1)		12 (9.4)		17 (9.3)	
	Nigeria	3 (5.5)		9 (7)		12 (6.6)	
	Syria	2 (3.6)		11 (8.6)		13 (7.1)	
	Yugoslavia	14 (25.5)		10 (7.8)		24 (13.1)	
	Other	19 (34.5)		60 (46.8)		79 (43.2)	
	Unknown	2 (1.09)		4 (2.19)		6 (3.28)	

### Traumatic events

For descriptive statistics, see Table 2. Overall in this sample, the trauma load was high, with each participant experiencing at least three traumatic events and a maximum of 14 events reported by five participants. Physical assault was most often reported in the total sample, followed by assault with a weapon, and combat or exposure to a war-zone (in the military or as a civilian). In the MI group, about 12.7% responded with “yes” to the statement “serious injury, harm, or death you caused to someone else” in comparison to 7.8% in the No-MI group. Fisher's exact test showed that this difference was not significant, *p* = 0.40. Furthermore, there were no significant differences in the endorsement of traumatic events between both groups, except for item 10 (“combat or exposure to a war-zone (in the military or as a civilian”) (94% in MI-group and 75% in No-MI group) and item 14 (“sudden violent death”) (80% in MI-group and 57% in No-MI group), respectively, *p* <0.005 and *p* <0.01.

**Table 2:** Number and percentage of the traumatic events reported by participants in each group.

**Measure**	**Moral injury group**		**No-moral injury group**		**Total**	
	***N***	***%***	***N***	***%***	***N***	***%***
Natural disaster	18	35.3	31	30.1	49	31.8
Fire or explosion	37	74.0	71	70.3	108	71.5
Transportation accident	28	54.9	64	62.7	92	60.1
Serious accident at work, home, or during recreational activity	15	29.4	26	25.5	41	26.8
Exposure to toxic substance	14	28.0	15	14.9	29	19.2
Physical assault	43	84.3	91	89.2	134	87.6
Assault with a weapon	44	86.3	88	98.1	132	86.8
Sexual assault	16	31.4	43	42.2	59	38.6
Other unwanted or uncomfortable sexual experience	12	23.5	37	35.9	49	31.8
Combat or exposure to a war-zone (in the military or as a civilian)^*^	48	94.1	77	74.8	125	81.2
Captivity	35	70.0	64	62.7	99	65.1
Life-threatening illness or injury	23	45.1	45	44.6	68	44.7
Severe human suffering	48	94.1	91	88.3	139	90.3
Sudden violent death^**^	41	80.4	59	57.3	100	64.9
Sudden accidental death	15	29.4	45	43.7	60	39.0
Serious injury, harm, or death you caused to someone else	7	14.0	8	7.7	15	9.7
Any other experience	24	51.1	51	50.0	75	50.3

* *p* <0.005 and

** *p* <0.01.

### Aim 1: Qualitative analyses of PMIEs

In total, all participants in the MI group reported at least one PMIE. Of the total sample (*N* = 55), 40 participants (72.7%) reported PMIEs that included commissions or omissions of themselves (MI-self), five participants (9.1%) reported PMIEs based on the acts and responsibility of others (MI-other), and six participants (10.9%) reported both. Furthermore, 21 participants (38.2%) reported events where they failed to act in a way that they found morally right (omissions), 27 participants (49.1%) reported acts with a moral transgression performed by themselves (commissions), and seven participants (12.7%) reported a combination of these two. Only six participants (10.9%) reported PMIEs that were related to being in combat as a soldier. The remaining participants reported PMIEs as civilians.

The descriptions of PMIEs contained mostly war related dilemmas and injuries and could be classified in the following categories: 1) failing to prevent harm to others (omission), 2) not giving aid to people in need (omission), 3) leaving family members behind that consequently lead to injury or death of others (commission), 4) making indirect and direct moral decisions that consequently led to injury or death of others (both commissions and omissions, 5) betrayal (commission) 6) and engaging in the harm of others (commission). The majority of the participants in this sample reported the fourth category, followed by the first category. Regarding the first category, participants mostly reported witnessing events of (extreme) violence and harm to others but failing to stop this violence. These events were accompanied by feeling powerless next to guilt, shame, and sadness. For the second category, the description that was mentioned most often was not giving medical help to others in need. Primarily, because they were injured themselves and therefore not able to help but feeling regret and guilt afterwards. As for the third theme, some participants reported that they left family members behind due to several reasons. Although it seemed the right decision at that moment, they reported feelings of guilt and regret, especially when they heard that the family members they left behind were in danger. The fourth category assembles a variety of events and was predominantly about the choice for a specific profession or the choice to become politically active, which caused a risk of arrestment or imprisonment or put others at risk. As for the fifth category, two participants reported events of betrayal. One person felt betrayed by others and the other person reported that he or she betrayed someone else under pressure and threat. Lastly, a few participants actively engaged in harming other people. Interestingly, almost everyone reported that they acted under duress because they were (physically) threatened.

### Aim 2: Quantitative analyses

#### PTSD severity

The vast majority of participants in this study met DSM-5 criteria for PTSD based on the CAPS (*N* = 160, 87.4%). Furthermore, 66 (36.1%) participants had a PTSD diagnosis with delayed expression and 39 (21.3%) participants had a PTSD diagnosis with dissociative symptoms. For descriptive statistics, see Table 3. The MI group reported greater PTSD severity and cluster D severity than the No-MI group, but a Welch *t*-test showed that this effect was not statistically significant for both PTSD severity and cluster D severity (Table 2). As the MI group included significantly more males than the No-MI group, an explorative one-way ANCOVA was used to examine if there was an effect of group (independent variable) on PTSD severity (dependent variable), whilst controlling for gender (covariate). Results showed no significant difference between the groups after controlling for gender, *F*_(1, 179)_ = 1.47, *p* = 0.22. For descriptive statistics, see Table 2.

**Table 3:** Descriptive statistics of PTSD severity symptoms and mental health symptoms.

**Measure**		**Moral injury**		**No-moral**		***t*_(180)_**	***p***	**Cohen's D**	**Total**	
		**group**		**injury group**						
		***M***	***SD***	***M***	***SD***				***M***	***SD***
PTSD severity		41.69	8.50	39.44	11.71	2.413	0.122	0.22	40.16	10.81
	Cluster B (severity)	11.56	3.37	11.23	3.92				11.33	3.74
	Cluster C (severity)	4.11	1.73	4.05	1.77				4.07	1.75
	Cluster D (severity)	14.46	3.79	13.58	5.08	1.837	0.177	0.24	13.86	4.71
	Cluster E (severity)	11.48	3.51	10.42	4.11				10.76	3.95
Mental health symptoms		2.29	0.66	2.19	0.75				2.22	0.72
	Somatic complaints	2.02	0.91	1.98	0.91				1.99	0.91
	Cognitive problems	2.59	0.74	2.59	0.93				2.59	0.87
	Interpersonal sensitivity	2.28	0.92	2.13	1.03				2.17	0.99
	Depressive mood	2.60	0.97	2.57	0.97				2.57	0.94
	Fear	2.56	0.84	2.46	0.90				2.49	0.88
	Hostility	1.78	1.02	1.57	1.03				1.64	1.02
	Phobic anxiety	2.20	1.00	1.94	1.06				2.03	1.04
	Paranoid thinking	2.44	0.97	2.22	1.05				2.29	1.03
	Psychoticism	1.94	0.80	1.98	0.93				1.97	0.89
	Feelings of guilt (item 52 BSI)	2.52	1.51	1.92	1.54				2.11	1.52

#### Mental health symptoms and feelings of guilt

The MI group reported slightly more mental health symptoms on the total BSI score than the No-MI group but this difference was not statistically significant *F*_(1, 168)_ = 0.63, *p* = 0.54. Also for the subscales of the BSI no statistically significant differences were found, all *F*_(1, 181)_ ≥ 0.013, all *p* ≥ 0.138. Based on the observation that the MI group included significantly more males than the No-MI group, an explorative one-way ANCOVA was conducted that examined the effect of group level on mental health symptoms (total BSI score), whilst controlling for gender. Results showed no significant difference between the groups after controlling for gender *F*_(1, 179)_ = 0.52, *p* = 0.47. On item level, the MI group reported significantly more feelings of guilt (item 52 of the BSI) than the No-MI group, *F*_(2, 167)_ = 4.02, *p* <005. For descriptive statistics, see Table 3.

## Discussion

The first aim of this study was to examine the nature of PMIEs among treatment seeking traumatized refugees in a qualitative manner. Over 30% of the refugees in this study reported one or more PMIEs at intake. The PMIEs of refugees included 1) failing to prevent harm to others, 2) not giving aid to people in need, 3) leaving family members behind that consequently lead to injury or death of others, 4) making indirect and direct moral decisions that consequently lead to injury or death of others, 5) betrayal, and 6) engaging in harming others. Failing to prevent harm to others, harming others and betrayal were described in earlier studies with military groups (16). However, the scope of PMIEs in refugees goes beyond combat-related PMIEs often found in the military. From the qualitative results it appeared that only 11% of the participants reported combat-related PMIEs that are similar to military personnel who were deployed in active duty.This study shed light on PMIEs that were specifically related to the refugee context, such as the decision to flee the country and leaving loved ones behind. In most cases the person felt guilt when they found out that those family members were harmed or persecuted, because of their decision to flee. The quantitative results showed that the MI-group reported significantly more often the experience of being in combat or exposure to a war-zone (measured with the LEC-5) than the No-MI group. This suggest that exposure to war or combat are important contextual factors in the experience of PMIEs in refugees.

These results provide insight into the difficult moral dilemmas and PMIEs that refugees can face. In contrast to earlier studies [e.g., (10)] the qualitative results of our study showed that the majority of refugees in the MI-group reported moral transgressions by themselves (MI-self) instead of transgressions by others, except for betrayal. Yet, it could be hypothesized that many identified moral transgressions in our study (e.g., failing to prevent harm to others) also involved transgressions by others, although this was not explicitly reported by the participants as a moral transgression (and therefore not reflected in the data). Also, the quantitative results of our study confirm that the MI-group was exposed to MI-Other experiences, reflected in their endorsement of items of the LEC-5. Here it was found that in the MI-group 80% of the participants witnessed a sudden violent death in comparison to 57% in the No-MI group. At least some of these deaths may involve moral transgressions by others (i.e., MI-Other experiences). Future research could investigate whether exposure to a war-zone and being witness to a sudden violent death are more likely to be experienced as morally injurious in comparison to other traumatic events. Interestingly, the MI-group included significantly more males than females in comparison to the No-MI group. This is comparable to other studies on moral injury in treatment seeking refugees (17). However, there is limited knowledge on gender differences in moral injury. The few studies available showed that PMIEs that included betrayal or being a witness were more often reported by women. No gender differences were found for perpetration-based PMIEs (34).

Our second aim was to compare refugees with and without PMIEs in terms of PTSD severity, feelings of guilt, and general mental health complaints. In contrast to our hypotheses, results showed no differences between the groups in terms of our outcome variables, except for feelings of guilt measured with one item of the BSI. This suggest that experiencing PMIEs is associated with more feelings of guilt but does not directly result in severe clinical symptoms. There are multiple possible explanations for our results. One explanation is that the refugees in this study were reluctant to provide details on experiences potentially yielding high levels of shame or guilt. As a result of human rights violations, mistrusting others can become a survival strategy for refugees in social contexts (35), reducing the chance of sharing sensitive details. Therefore, PMIEs may be underreported at intake, which is before treatment and before a trusting therapeutic alliance has been established. Hence, a number of refugees may be incorrectly assigned as No-MI because the PMIEs were rated by clinicians at intake and no specific measure of PMIEs was administered. Also ceiling effects might play a role since both groups consisted of severely traumatized individuals. Another explanation for these findings could stem from the difference between MI-self and MI-other. It has been postulated that facing moral violations of others is associated with life-threat and fear, resulting in more PTSD symptoms, in comparison to moral violations of oneself which is more associated with guilt and shame (20). In this study, the majority of the participants (72.7%) reported PMIEs that included moral transgressions of oneself. This suggests that guilt was more dominant than fear, perhaps resulting in less elevated PTSD symptoms than expected. It might be possible that committing a moral transgression is related to different outcomes compared to witnessing a moral transgression. It would be interesting to investigate whether omissions and commissions have different outcomes in terms of mental health symptoms. This is relevant for the treatment of distress associated with moral injury. For refugees in specific, this study acknowledges the importance of focusing on cognitive evaluations regarding responsibility, failing to prevent harm to others and decision making, as these were the most important themes that resulted from the qualitative analyses. Considering that our study showed that feelings of guilt were significantly stronger in the MI-group compared to the No-MI group, interventions that address guilt are also advised. For instance, Trauma-Informed Guilt Reduction (TrIGR) is a transdiagnostic psychotherapy that addresses guilt, shame, and moral injury symptoms after exposure to PMIEs and is indicated for a variety of trauma types including exposure to war and combat (36, 37). Also, the Brief Eclectic Psychotherapy for Moral Trauma (BEP-MT) is a newly developed treatment protocol that integrates components of cognitive-behavioral, psychodynamic, and systemic psychotherapy and was researched in a single case study (38).

A strength of the study is that it is the first that qualitatively examined the type of PMIEs experienced by treatment-seeking refugees with PTSD symptoms. Nevertheless there are several limitations to this study. The first limitation is that PMIEs were identified based on information obtained during intake sessions. No specific measure of PMIEs was administered. As a result it is possible that participants were incorrectly categorized as No-MI or vice versa. In this study, those participants for whom a distinction could not be made or data were missing in order to make a decision, were excluded, resulting in a considerable reduction of the sample size. Consequently, it is plausible that important information is missed. Also, the clinicians that made the categorizations of PMIEs pre-selected events that focused on a perceived moral decision or moral conflict by the person himself. However, this might unintentionally resulted in mainly MI-self experiences instead of MI-other experiences, which could explain why mainly MI-other themes were revealed in the qualitative analyses. Future studies should examine PMIEs more systematically. Furthermore, all of the participants were treatment seeking, which reduces the generalizability of the findings. Another limitation is the cross-sectional design of the study, lacking information on the course of mental health of participants over time, which would provide a more comprehensive understanding on the development of mental health complaints in relation to PMIEs. Finally, only guilt was taken into account whereas other emotions such as blame, regret, shame or anger are also important outcome measures of PMIEs. Also, it is a lack of this study that guilt was only measured with one item and not with a validated instrument.

In conclusion, this study illustrates the presence of PMIEs in a refugee population. Refugees with one or more PMIE had more feelings of guilt in comparison to refugees with no PMIEs but scores on indices of PTSD and general psychopathology were similar in the two groups. Further research needs to look into the PMIEs of refugees with a valid instrument to assess moral injury in a large sample and monitor PTSD complaints over time. Furthermore, the differences between commissions and omissions and moral transgressions performed by oneself or others remains unclear. Future studies should investigate this in order to understand the relationship between PMIEs and mental health outcomes in refugees.

## Data availability statement

The data analyzed in this study is subject to the following licenses/restrictions: Due to the nature of this research, participants of this study did not agree for their data to be shared publicly, so supporting data is not available. Requests to access these datasets should be directed to databeheer@arq.org.

## Ethics statement

Ethical review and approval was not required for the study on human participants in accordance with the local legislation and institutional requirements. The patients/participants provided their written informed consent to participate in this study.

## Author contributions

SR contributed to the study concept and design. NM analyzed the data and drafted the manuscript. SR, NM, and PB participated in the interpretation and revision process of the manuscript. All authors read and approved the final manuscript.

## Conflict of interest

The authors declare that the research was conducted in the absence of any commercial or financial relationships that could be construed as a potential conflict of interest.

## Publisher's note

All claims expressed in this article are solely those of the authors and do not necessarily represent those of their affiliated organizations, or those of the publisher, the editors and the reviewers. Any product that may be evaluated in this article, or claim that may be made by its manufacturer, is not guaranteed or endorsed by the publisher.
